# Copper Oxide Electrochemical Deposition to Create Antiviral and Antibacterial Nanocoatings

**DOI:** 10.1021/acs.langmuir.4c00642

**Published:** 2024-07-09

**Authors:** Anna Kusior, Julia Mazurkow, Piotr Jelen, Maciej Bik, Sada Raza, Mateusz Wdowiak, Kostyantyn Nikiforov, Jan Paczesny

**Affiliations:** † AGH University of Krakow, Faculty of Material Sciences and Ceramics, Mickiewicza 30, Kraków 30-059, Poland; ‡ Institute of Physical Chemistry, Polish Academy of Sciences, Kasprzaka 44/52, Warszawa 01-224, Poland

## Abstract

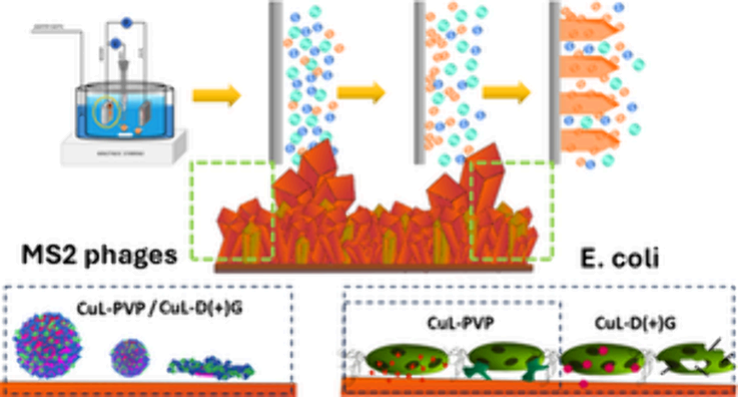

The impact of the reaction environment on the formation of the polycrystalline layer and its biomedical (antimicrobial) applications were analyzed in detail. Copper oxide layers were synthesized using an electrodeposition technique, with varying additives influencing the morphology, thickness, and chemical composition. Scanning electron microscopy (SEM) images confirmed the successful formation of polyhedral structures. Unmodified samples (CuL) crystallized as a mixture of copper oxide (I) and (II), with a thickness of approximately 1.74 μm. The inclusion of the nonconductive polymer polyvinylpyrrolidone (PVP) during synthesis led to a regular and compact CuO-rich structure (CuL-PVP). Conversely, adding glucose resulted in forming a Cu_2_O-rich nanostructured layer (CuL-D(+)G). Both additives significantly reduced the sample thickness to 617 nm for CuL-PVP and 560 nm for CuL-D(+)G. The effectiveness of the synthesized copper oxide layers was demonstrated in their ability to significantly reduce the T4 phage titer by approximately 2.5–3 log. Notably, CuL-PVP and CuL-D(+)G showed a more substantial reduction in the MS2 phage titer, achieving about a 5-log decrease. In terms of antibacterial activity, CuL and CuL-PVP exhibited moderate efficacy against *Escherichia coli*, whereas CuL-D(+)G reduced the *E. coli* titer to undetectable levels. All samples induced similar reductions in *Staphylococcus aureus* titer. The study revealed differential susceptibilities, with Gram-negative bacteria being more vulnerable to CuL-D(+)G due to its unique composition and morphology. The antimicrobial properties were attributed to the redox cycling of Cu ions, which generate ROS, and the mechanical damage caused by nanostructured surfaces. A crucial finding was the impact of surface composition rather than surface morphology on antimicrobial efficacy. Samples with a dominant Cu_2_O composition exhibited potent antibacterial and antiviral properties, whereas CuO-rich materials showed predominantly enhanced antiviral activity. This research highlights the significance of phase composition in determining the antimicrobial properties of copper oxide layers synthesized through electrodeposition.

## Introduction

The effect of copper on viruses has been studied for decades. Since 1956, after Silver et al. reported the antiviral activity of the copper ions,^1^ subsequent studies devoted to copper-based materials and their defense against a range of viruses, including poliovirus,^2^ human immunodeficiency virus type 1 (HIV-1),^3^ and West Nile virus (WNV),^4^ have been published. Furthermore, synergistic interactions between copper and free chlorine have been observed to inactivate bacteriophages such as MS2,^5^ PhiX174, Phi6, and T7.^6^ Salah et al. emphasized the role of reactive oxygen species (ROS) generation and ion release from copper surfaces in virus inactivation, particularly against SARS-CoV-2, influenza A, and murine norovirus.^7^

In addition to their antiviral properties, copper and copper-based nanoparticles have garnered attention for their antibacterial efficacy. The significant advantage of copper-based materials, compared to popularly used silver nanoparticles,^8^ is the significantly lower cost of the material. The potential of copper and copper-based nanoparticles in medicine was recently described in review articles by Woźniak-Budych et al.,^9^ Bisht et al.,^10^ and Li et al.^11^ In some studies, copper-based nanoparticles surpassed silver nanoparticles as antibacterial agents.^12^ Other reports proved the synergistic action of copper or copper oxide (II) and silver nanoparticles.^13,14^

The mechanisms of potential cytotoxicity primarily revolve around contact killing and the controlled release of active agents, including ions and reactive oxygen species (ROS). There have been studies to measure superoxide anion production using an NBT-light system (nitroblue-tetrazolium). CuO reduces superoxide anion levels by reacting with O^2-·^, forming Cu(I). Furthermore, hydroxyl radical production assessed via the reduction of deoxyribose shows higher OH^–·^ levels for CuO at lower concentrations, facilitated by intracellular H_2_O_2_ conversion through Fenton-like reactions.^15^ However, concerns regarding the toxicity of copper-based agents, including nanoparticles, have been raised.^16,17^

The concerns surrounding the toxicity of copper-based agents and the potential development of bacterial resistance seem to be partially eliminated using nanocoating and not suspensions of nanoparticles. First, the active agent is securely immobilized on the surface, reducing mobility, lowering the risk of exposure (e.g., by inhalation) or contamination, and increasing stability. Second, precisely applying these coatings can significantly curtail the release of ions or ROS and limit interactions with other organisms, such as in air filters. Lastly, it is worth noting that the antibacterial effect is localized, with the primary objective being the prevention of biofilm formation, as opposed to the traditional antibiotic approach. However, most antiviral agents interfere with various stages of the viral replication cycle related to the host cell (i.e., recognition of the host, infection, multiplication, or release of progeny virions).^18^ However, such an action is required for nanocoating to be efficient in preventing the spread of pathogens, primarily via contact with exposed surfaces, e.g., doorknobs, handles in public transportation, etc.

The surface of an object has properties different from those of bulk material. However, only a limited number of materials have surfaces with desired properties. To address this, surface modification techniques have emerged as indispensable tools for tailoring surface properties to meet specific criteria. Surface nanoengineering leads to structures with a high percentage of their constituent atoms at a surface, enhancing the effect.

Among surface modification techniques, electrochemical methods stand out for their precision and control, *in situ* monitoring, tunable and tailored material properties, uniformity of obtained surfaces, versatility, compatibility with existing technologies, and cost-effectiveness.^19,20^ Considering this, we developed a new method for depositing Cu_2_O layers tailored for biomedical applications. Whereas previous studies primarily focused on the photocatalytic properties of Cu_2_O,^21−24^ our investigation explores the potential use of Cu_2_O and CuO coatings as promising antibacterial and antiviral properties.

In this work, we have studied the additives’ effect on the electrodeposited copper oxide layers. The impact of the reaction environment on the formation of the polycrystalline layer and its surface properties were analyzed in detail, focusing on their possible use for biomedical (antimicrobial) applications. Employing 3D Raman imaging, the distribution of copper(I) and (II) oxides was determined, which made it possible to establish the actual influence of the materials on their activities.

## Results and Discussion

Electrodeposition is one of the oldest and most environmentally friendly methods for producing nanostructured layers, mainly nanowires, nanotubes, or particles with well-defined facets.^25−27^ So far, Cu_2_O layers have been obtained by applying different electrolytes, surfactants, and pH adjustment.^28^ This significantly affected the form of the grains and their packing. Polyhedral shapes and compact oxide layers have also been synthesized by complexing copper ions.^29^ This methodology made it possible to modify the surface properties of Cu_2_O using additional agents directly.

**Scheme 1:**
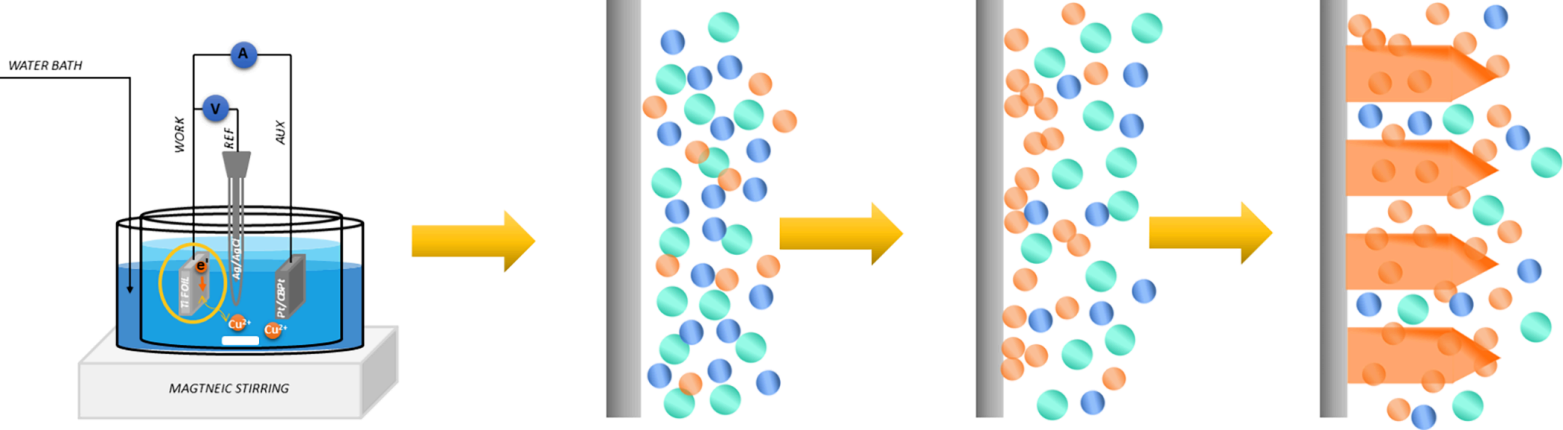
Schematic Illustration of the Adsorption Process on the Electrodes and Formation of the Cu_2_O Nuclei The blue, green, and orange circles represent OH^–^ and copper ion complexes, acid ions, and the resulting copper oxide molecules.

Herein, three different variants of coatings were prepared to analyze the effect of the additives on the polyhedral copper oxide formation (Scheme 1): one by supplementing the nonconductive polymer poly(vinylpolypyrrolidone) (PVP) (CuL-PVP), the second by using the strong reductant in the form of d(+) glucose (CuL-D(+)G), and the last one as a reference without any additive (CuL). The cupric sulfate and lactic acid solution were electrolytes in all three cases. Because of the complexing of the lactic ions (L^+^), the Cu^2+^ ions are stabilized.^29^ Moreover, the L^+^ can be selectively adsorbed at the [111] planes, and thus, the [100] plane can grow faster. On the other hand, PVP adsorbs on highly active surfaces (high index),^30^ which should block the growth of the mentioned plane [100]. The addition of glucose aimed to reduce or maintain low levels of elemental oxidation. The detailed synthesis parameters are summarized in Table 1.

**Table 1:** Electrochemical Bath Conditions and the Sample Abbreviation^a^

**sample**	**CuSO**_**4**_**(g)**	**chelating agent**	**H**_**2**_**O (mL)**	**capping agent**	**reducing agent**	**conditions**		
		**lactic acid (mL)**		**PVP (g)**	**C**_**6**_**H**_**12**_**O**_**6**_**(g)**	***T***_**b**_**(°C)**	***t***_**d**_**(min)**	***V***_**B**_**(mV)**
CuL	5	12.5	37.5	0.5	0.5	60	30	750
CuL-PVP	5	12.5	50.0					
CuL-D(+)G	5	12.5	37.5					

a *T*_b_: temperature of the bath, *t*_d_: deposition time, *V*_p_: applied potential.

**Figure 1:**
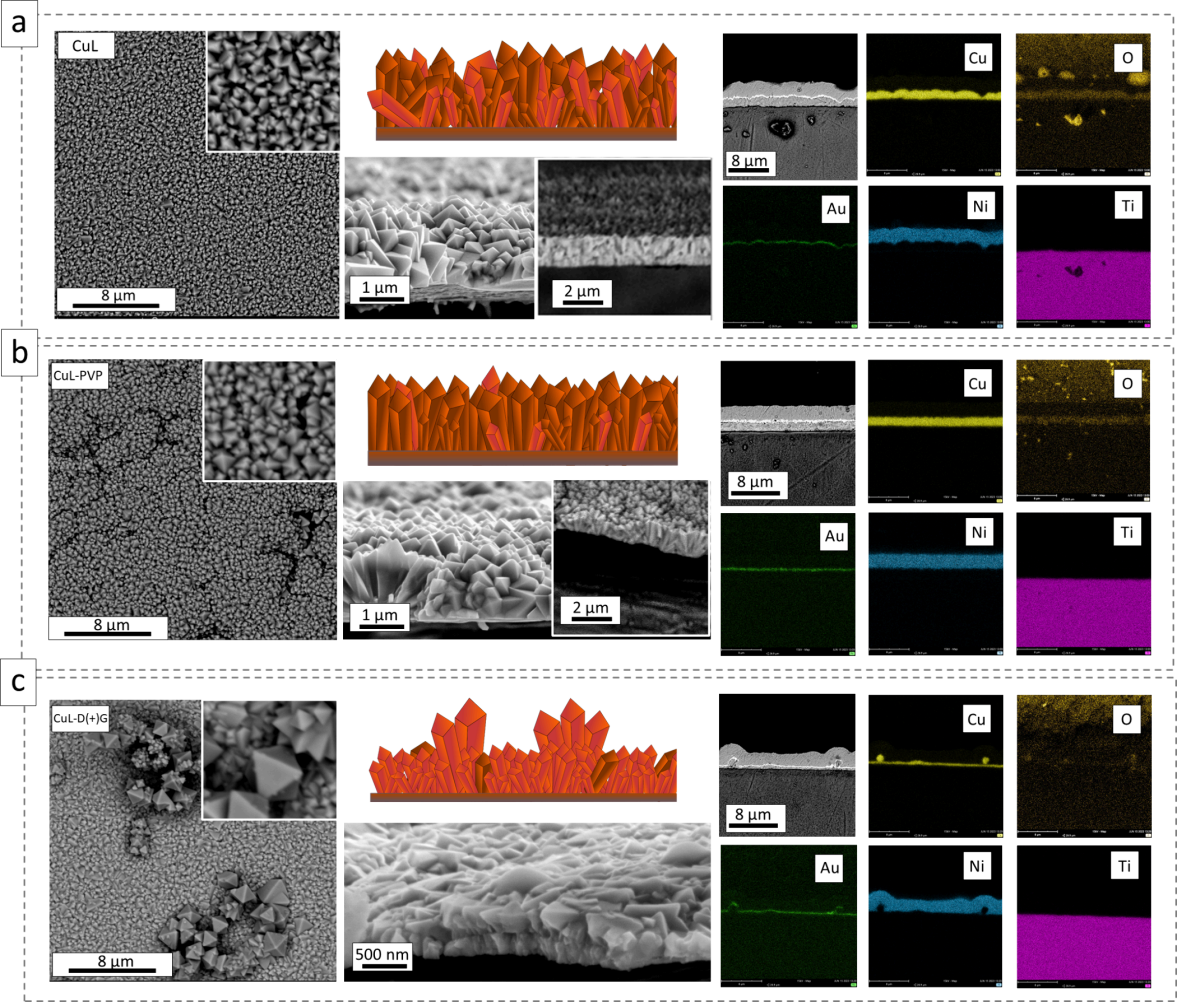
SEM images of the deposited (a) CuL, (b) CuL-PVP, and (c) CuL-D(+)G copper oxide layers (top view and side view) with the cross-sectional microstructure EDS analysis.

Figure 1 shows the SEM images of the copper oxide layers grown by electrochemical deposition. Polyhedral materials were obtained according to the assumptions of material synthesis. The crystal-like particle packing differed depending on the agent used during the synthesis. In the initial stage of the process, copper ions in contact with lactic acid form CuL_2_^2–^ and [CuL_2_(OH)]^3–^ complexes. Afterward, they are reduced to form copper oxide (I), which is adsorbed at the electrode surface and marks the beginning of the nucleations.

Whereas no additional substances are present in the solution (CuL), the received sample shows homogeneously deposited elongated crystal-like forms with an overall thickness of about 1.74 μm (Figure 1a). When PVP is added (CuL-PVP), no significant changes are visible from the top of the view. However, a decrease in sample thickness (617 nm) and regular alignment of the polyhedral are observed. The inhibition of the structure’s growth indicates oppositely acting mechanisms related to L^+^ ions and the polymer chain (Figure 1b). For samples obtained assisted by glucose (CuL-D(+)G), the large elongated forms above the surface are visible. That may be due to the effect of the reductant itself on the precipitation of Cu_2_O directly in the solution, which undergoes electrophoretic deposition as a result of the applied potential due to the internal stress arising during grain growth (Figure 1c). The average layer thickness determined by SEM cross-section analysis is approximately 560 nm. The chemical composition of the layers was confirmed by the EDS analysis presented in Figure 1 and Figure S1, whereas the data are summarized in Table S1.^29^

**Figure 2:**
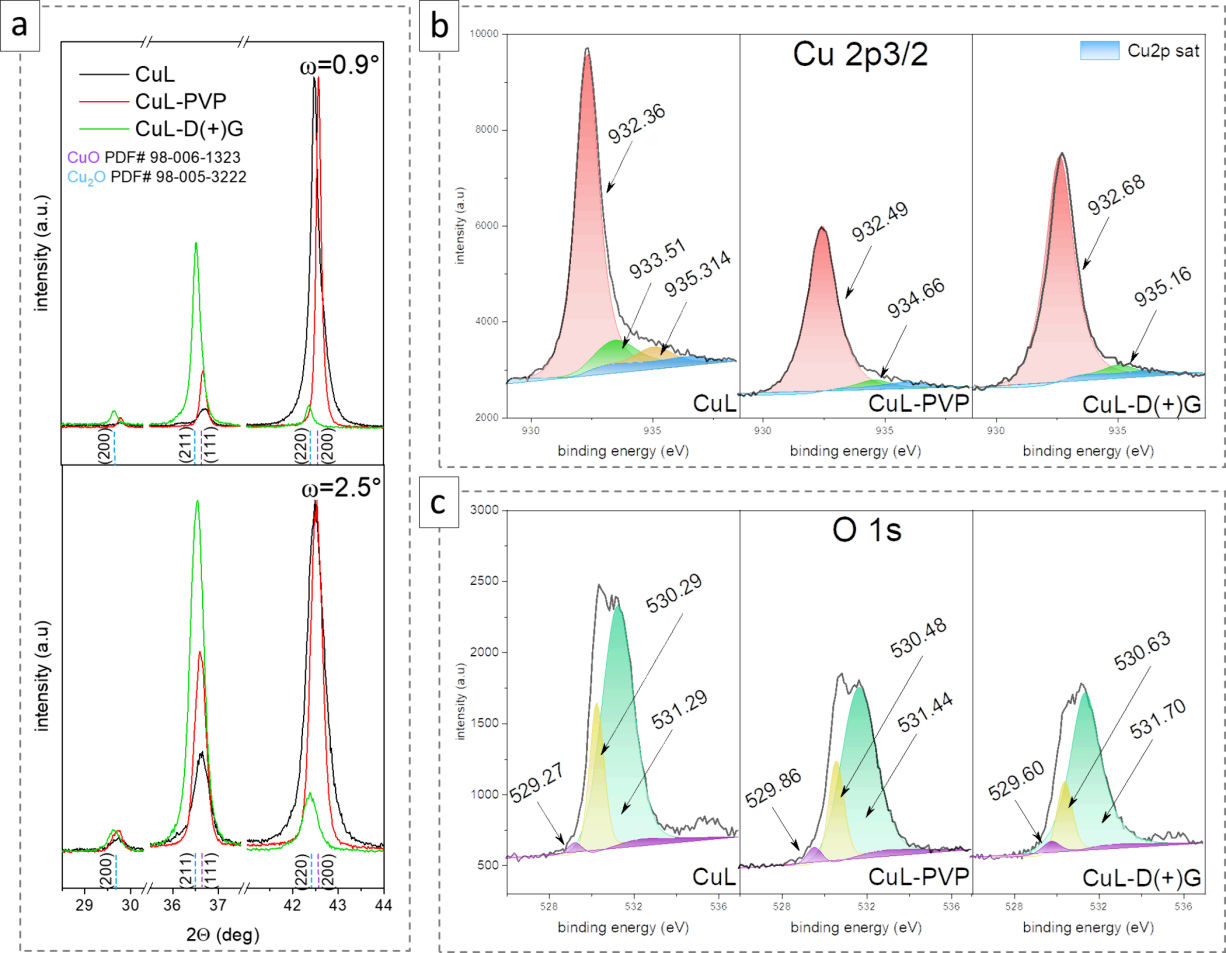
XRD analysis of the deposited CuL, CuL-PVP, and CuL-D(+)G copper oxide layers (a) in the Bragg–Brentano and GID configuration. XPS examination of Cu (b) and O (c) surface chemical bonding states.

Copper-based compounds are known for their antimicrobial properties. However, in contrast to Cu_*x*_O, Cu particles are relatively unstable. This paper aimed to receive samples with well-defined facets, which could affect the activity of the materials but also fully oxidized copper-based forms. According to the XRD pattern (Figure 2a), all films are polycrystalline and can be indexed to cubic forms of CuO (PDF# 98-006-1323, *Fm-*3*m*) and Cu_2_O (PDF# 98-005-3222, *Pn*-3*m*). However, the ratios differed despite adjusting the solution to pH = 10. According to the GID analysis at ω = 0.9°, the dominant phase for CuL and CuL-PVP is CuO, with about 74.5 and 81.1%, respectively. The increase of the ω to 2.5° results in a change in the proportion of Cu_2_O, from 12.7 to 25.5% for CuL and from 7.5 to 18.9% for CuL-PVP. That suggests that the materials are biphasic. However, the reduced form is present in the volume of the material, whereas the material is oxidized only at the layers’ surface. On the other hand, the CuL-D(+)G sample seems to consist only of Cu_2_O (Table S2). Adding a reducing agent (glucose) induced the growth of copper oxide (I). So far, which form of the oxide is most favorable for biomedical applications has yet to be understood, as both exhibit antimicrobial capabilities. However, given the possibility of generating ROS, their mixture should show increased activity.

To more broadly characterize the surface state of the oxide layers, an XPS test was carried out to confirm the surface chemical composition and oxidation states (Figure 2b,c). As shown in Figure 2b, the main Cu peak (with an asymmetric tail) is present at positions 932.36, 932.49, and 932.68 eV for CuL, CuL-PVP, and CuL-D(+)G, respectively. It was assigned to Cu 2p_3/2_ of Cu^1+^.^31,32^ The recorded signal at 933.51 and 935.31 eV for Cu-L corresponded to copper oxide (II) and hydroxide, respectively. The detection of shakeup satellites between 940 and 947 eV denoted the presence of Cu^2+^ 2p_3/2_. In the other two samples, the observed signals at 934.66 eV (CuL-PVP) and 935.16 eV (CuL-D(+)G) were weak. However, it did not exclude the appearance of the copper-based compounds.^33^ The chemical states of the sulfur atoms assigned to the S 2p_3/2_ and 2p_1/2_ in the elementary SO_4_^2–^ form (Table S3) were confirmed only in the CuL-PVP and CuL-D(+)G samples.^33^ The corresponding O 1s spectra (Figure 2c) show three types of oxygen related to Cu–O–Cu, Cu–O defects, and Cu–OH, CO.

**Figure 3:**
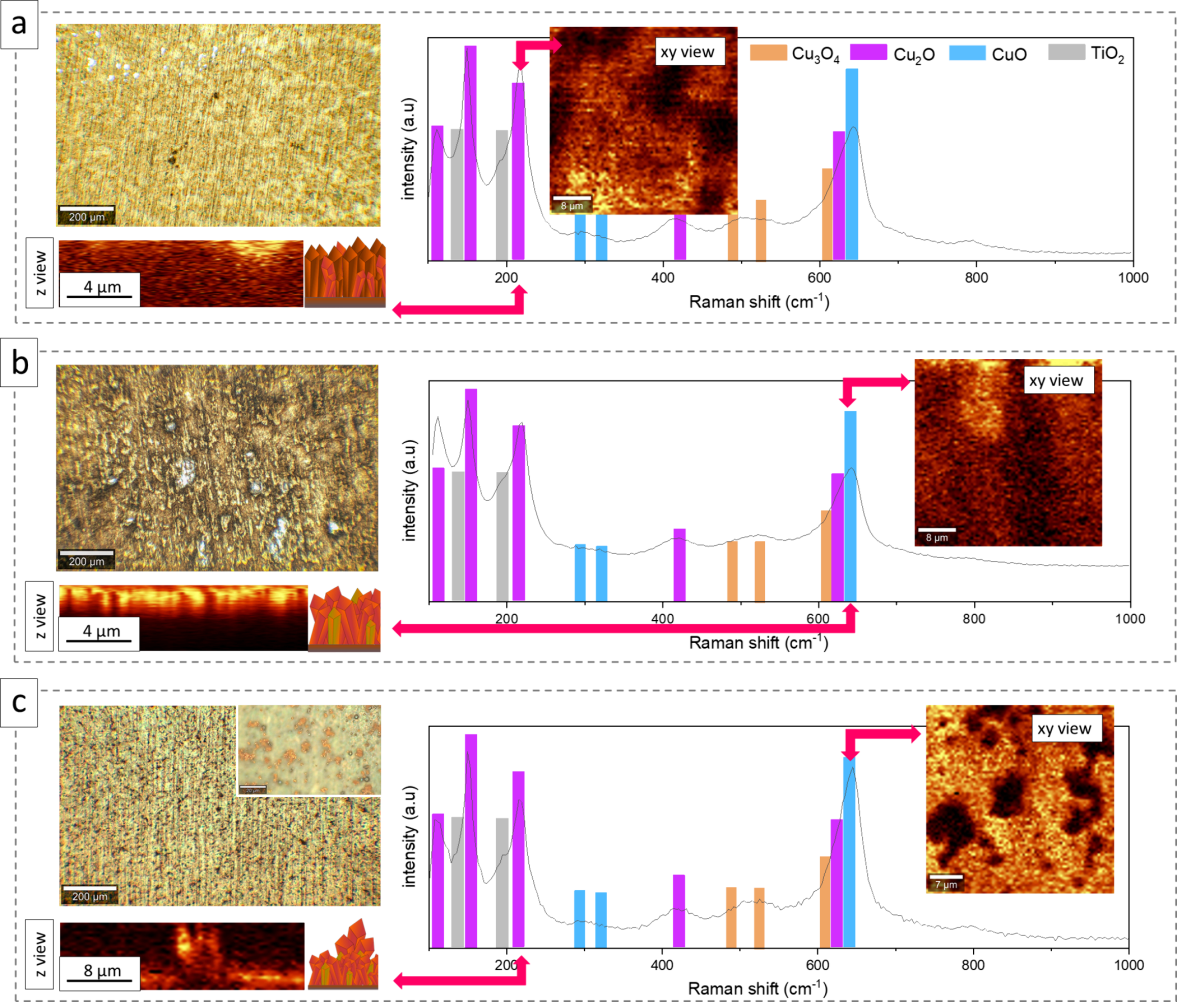
Raman imaging of the deposited (a) CuL, (b) CuL-PVP, and (c) CuL-D(+)G copper oxide layers, cross sections (*z* direction), and surface map (*xy* view) of the assigned band position.

Another technique to determine the surface composition of samples, crucial in the context of antibacterial properties, is Raman spectroscopy. Figure 3 shows the Raman spectra of the deposited copper oxide films with the corresponding intensity maps in the *xy* view and *z* direction assigned to the specific band positions. All samples presented characteristic phonon frequencies of the crystalline Cu_2_O (112, 149, 217, and 628 cm^–1^) and CuO (296, 320, and 638 cm^–1^)^34^ forms. The obtained results were in agreement with the XRD data. More detailed information was collected by 3D scanning using Raman spectroscopy. For CuL, the presence of the most intense 217 cm^–1^ band at the surface (*xy* view) and at the different *z* high confirmed the dominant share of copper(I) oxide in the whole volume of the layer. A more equal phase distribution was found in the case of CuL-PVP. The 638 cm^–1^ vibration mode analysis showed that the copper(II) oxide crystallized mostly at the sample surface. Similar observations were made according to CuL-D(+)G; however, the largest polyhedral growing above the surface was rich in Cu_2_O. Moreover, analysis of mode intensities related to CuO (Figure S3) suggested various crystal orientations in this sample.

Our research also revealed additional modes related to Cu_3_O_4_ (419, 321, and 624 cm^–1^) in all samples. The vibrating bands reported at 144 and 191 cm^–1^ might be attributed to the anatase (TiO_2_) at the substrate’s surface. Notably, the results confirmed that the phase variation of copper oxides on the surface of materials was highly dependent on the reaction environment in which electrodeposition was carried out. This complex interplay between the reaction environment and the resulting surface composition of copper oxides is a critical factor in objectively evaluating the antimicrobial properties, adding a layer of depth to our research.

### Antiviral and Antibacterial Efficacy

The antiviral properties of nanocoatings were tested against bacteriophages T4 and MS2. Phages are a major threat to processes utilizing bacteria to produce active substances (i.e., insulin is produced in *Escherichia coli*(35)). MS2 belongs to Leviviricetes, consisting of a capsoid in which genetic material (RNA) is stored. and its morphology is much simpler. MS2 is considered a good surrogate for studies of viruses infecting eukaryotic cells.^36^ It was used as a model to study COVID-19,^37,38^ human norovirus,^39^ and other enteric viruses.^40^ It is also a challenging target, as it can withstand the action of agents deactivating other phages.^41^ We chose T4 (Caudoviricetes) as it was found that most phases (more than 96%) have similar morphology (i.e., tailed phages).^42^ It comprises a capsid (protein shell with DNA inside), a tail, and fibers. T4 is a good model for studying antiphagents (i.e., antibacteriophage agents), which is crucial from the biotechnology point of view, where active compounds are produced in bacteria.^36^

**Figure 4:**
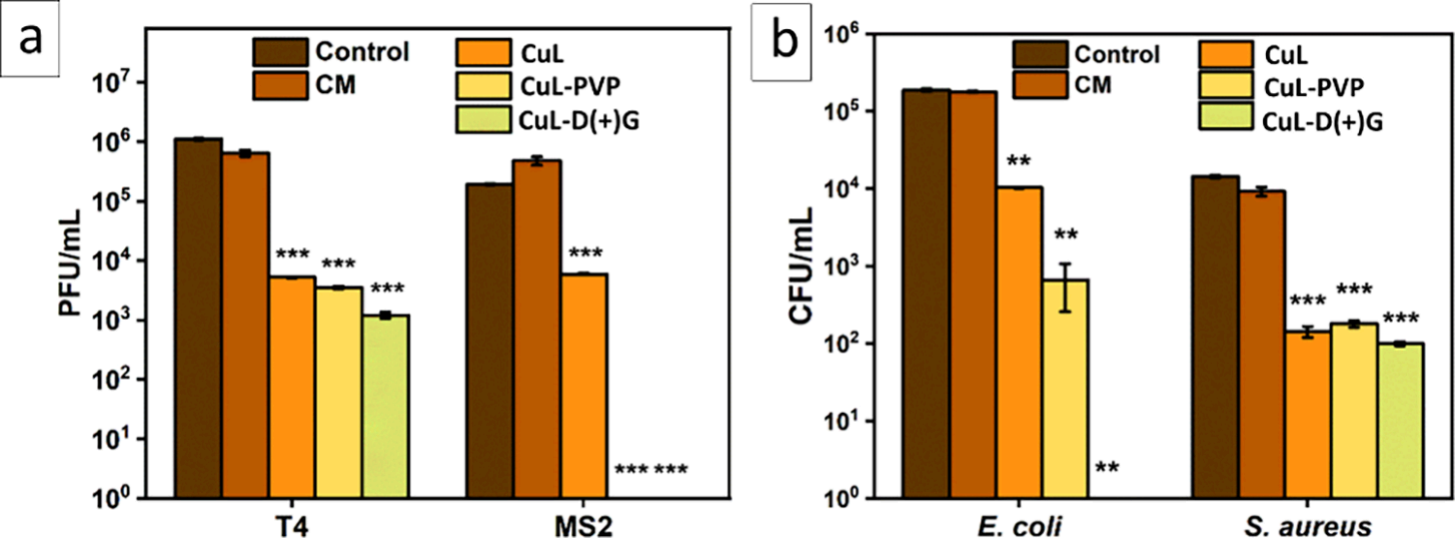
Evaluation of the biological activity of copper oxide nanocoatings. (a) The antiviral activity of CuL, CuL-PVP, and CuL-D(+)G against T4 and MS2 bacteriophages. The results were presented as plaque-forming units per milliliter (PFU/mL). (b) The antibacterial activity CuL, CuL-PVP, and CuL-D(+)G against *E. coli* and *Staphylococcus aureus*. The results were presented as colony-forming units per milliliter (CFU/mL). CM stands for control material, i.e., unmodified substrate, and control is the suspension of phages or bacteria without anything added to it. Statistical analysis was performed using Student's *t* test with respect to CM: * *p* < 0.05; ** *p* < 0.01; *** *p* < 0.001.

The phages were exposed to the pieces of titanium foil coated with CuL, CuL-PVP, and CuL-D(+)G. The pieces were of identical size. As controls, we analyzed phage suspension and phage suspension exposed to the substrate (titanium foil). The samples were stirred at room temperature for 4 h, and the differences in the number of PFU/mL were recorded using a droplet test on double-layer LB-agar plates. The results are presented in Figure 4a. All the experiments were performed in triplicate.

Unmodified substrates did not cause any significant decrease in the number of active virions. For the T4 bacteriophage, the activity of CuL, CuL-PVP, and CuL-D(+)G materials was similar (about a 2.5–3-log decrease in phage titer), with an emphasis on CuL-D(+)G, which performed slightly better than other materials. In the case of MS2, the antiviral activity of CuL material was similar to that of T4 phage, resulting in the reduction of phage titer by about 2.5 log. However, for CuL-PVP and CuL-D(+)G, the decrease in phage titer was below the methods’ detection limits (about 25 PFU/mL), i.e., by around 5 log.

This antiviral activity may be explained by implementing the additives during the electrodeposition of copper oxide. In the case of CuL-PVP and Cu-D(+)G, the materials show the dominance of one phase, CuO and Cu_2_O, respectively. The CuL sample is a mixture of both phases. The results suggest a drift of phase properties that may dominate the other two. The literature reports show that the presence of PVP might alternate with the phage protein profile.^43,44^ On the other hand, CuL-D(+)G might accelerate damage by ROS and copper ions.

Next, we tested the antibacterial activity of studied coatings against representative strains of Gram-negative (*E. coli*) and Gram-positive (*S. aureus*) bacteria. Gram-negative and Gram-positive bacteria have different physical factors susceptibilities due to differences in the cell envelope morphology,^45^ i.e., the thickness of the cell wall. The microbes were exposed to covered pieces of foil at room temperature for 4 h upon stirring, and the differences in the number of CFU/mL were recorded using the plating method. The results are presented in Figure 4b. The differences in the antibacterial activity were observed in the antibacterial assay. For *E. coli*, the exposure to CuL material resulted in about a 1-log decrease in the titer. The CuL-PVP material provided better antibacterial activity, causing a 2-log decrease in bacterial titer. In contrast, the CuL-D(+)G material caused a decrease in *E. coli* titer to below the methods’ detection limits (about 10 CFU/mL), i.e., by around 5 log. Surprisingly, in the case of *S. aureus*, all the materials provided similar antimicrobial activity, causing an approximately 2.5-log decrease in bacterial titer. For both bacterial species, exposure to nonmodified material control material (CM) did not cause a significant decrease in the titer.

Usually, Gram-negative bacteria are more resistant to toxic molecules, such as antibiotics, digestive enzymes, detergents, heavy metals, and dyes. The lipopolysaccharide (LPS) layer acts as a scavenger.^46^ Therefore, Gram-positive bacteria proved to be more vulnerable to the release of ions.^47^ This was true in our case for CuL and CuL-PVP. The opposite results obtained for CuL-D(+)G suggested other mechanisms involved in the case of this material. This might originate in the differences in the composition and morphology of the CuL-D(+)G coatings. CuL-D(+)G was synthesized in glucose, forming large polyhedral crystals above the surface, which were rich in Cu_2_O (cf. Figures 1 and 2). First, it was proven that Cu(I) is more toxic than Cu(II) to cells of *E. coli*. The tests were performed under anaerobic conditions to maintain the stability of Cu(I).^48^ Second, numerous studies have concentrated on examining the capacity of Cu ions to undergo redox cycling between Cu^+^ and Cu^2+^. Such a Fenton-like reaction may produce reactive oxygen species, instigating lipid peroxidation, protein oxidation, and DNA damage.^49^ Liquid peroxidation might differentiate between Gram-negative and Gram-positive bacteria, as the latter do not have external cell membranes. Finally, we showed previously that Gram-negative bacteria are much more vulnerable to mechanical damage due to sharp, rod-like nanoparticles.^47^ This is because of a much thinner cell wall than Gram-positive bacteria (1.5–10 vs 20–80 nm^45^).

## Conclusions

Copper oxide layers were synthesized using the electrodeposition technique. Implementing different additives influences the form, sample thickness, and chemical composition. SEM images revealed that polyhedral materials were successfully obtained. Unmodified samples (CuL) crystallize in a mixture of copper oxide (I) and (II), with a thickness of about 1.74 μm. The presence of the nonconductive polymer (PVP) during synthesis affects the formation of a regular and compact CuO-rich structure (CuL-PVP). Adding glucose influences the formation of a Cu_2_O-rich nanostructured layer (CuL-D(+)G). In both cases, the modifiers reduce the sample thickness to 617 and 560 nm, respectively. All coated materials reduced the T4 phage titer by about 2.5–3 log, whereas CuL-PVP and CuL-D(+)G showed a more significant reduction in MS2 phage titer (about 5 log). CuL and CuL-PVP exhibited modest antibacterial activity against *E. coli*, whereas CuL-D(+)G reduced the titer to undetectable levels. All materials caused similar reductions in *S. aureus* titer. The presence of PVP and glucose significantly influenced the antimicrobial efficacy, likely due to phase properties and the generation of reactive oxygen species (ROS).

Differences in susceptibility were noted, with Gram-negative bacteria being more vulnerable to CuL-D(+)G due to its unique composition and morphology. The antimicrobial properties are linked to the ability of Cu ions to undergo redox cycling, generating ROS, and the mechanical damage caused by the nanostructured surfaces. A key aspect of the research turned out to be the influence not so much on the surface shape of the materials but on their phase (surface) composition. A sample with a dominant Cu_2_O composition shows good antibacterial and antiviral properties, whereas a CuO-rich material shows only increased antiviral activity.

## Experimental Section

### Materials

Titanium foil (Ti, 99.7% trace metal basis, thickness 0.127 mm) was purchased from Sigma-Aldrich. Lactic acid 88% (Chempur), anhydrous d-(+)-glucose (POCH), polyvinylpyrrolidone (Mw 40,000, Alfa Aesar), CuSO_4_·5H_2_O (EuroChem), and sodium hydroxide (POCH) were used toward the copper oxide layer synthesis.

The liquid LB medium contained 10 g/L NaCl, 10 g/L tryptone, and 5 g/L yeast extract. LB-agar was additionally supplemented with 15 g/L of agar. The Top-LB agar was supplemented with 7.5 g/L of agar. All the media were purchased as premix (Carl Roth, Germany). For bacteriophage suspension dilutions, a TM buffer (10 mM Tris base, 5 μM CaCl_2_, 10 mM MgSO_4_; all components were purchased from Sigma-Aldrich (USA)) was used. For bacteria suspension dilutions, a 0.9% solution of NaCl (Sigma-Aldrich, >99%, estimated by AgNO_3_ titration) in Milli-Q water was used.

### Material Preparation

Copper oxide films were electrodeposited on a metallic substrate in a three-electrode configuration cell, where a platinum wire was used as a working electrode, Ag/AgCl as a reference, and titanium foil as a counter electrode. First, Ti (1.5 × 2 cm) was cleaned with acetone and ethanol and rinsed with deionized water (DIW). Afterward, to remove the naturally formed titanium oxide layer, the substrates were immersed in 35% HCl for a few minutes and rinsed with DIW. The electrolytic bath contained 0.4 M copper(II) sulfate (CuSO_4_·5H_2_O) and 3 M lactic acid ([CH_3_CH(OH)COO]_2_Ca·H_2_O) as a chelating agent. To modify the crystal’s growth pathway, 0.5 g of glucose (C_6_H_12_O_6_) or PVP was added as the oxidizing compound and the surfactant, respectively. The pH of the solution was adjusted to 10 by 4 M NaOH. All procedures were carried out at approximately 60 °C in a water bath. Before the synthesis, foils were cyclically scanned from −1 to 1.3 V with inversely coupled counter and working electrodes. Deposition of the copper oxide layer lasted 30 min at the potential of 750 mV and was preceded by holding the sample in the bath for 20 min. The obtained layers were rinsed with DIW and dried in the air at room temperature.

### Material Characterization

The coatings’ cross-sectional observations (side views) were done using a ThermoFisher Scientific Apreo 2 scanning electron microscope. Moreover, the “coating” surface, cross-sectional microstructure, and chemical composition were examined with a Thermo Fischer Scientific Phenom XL Desktop scanning electron microscope with an electron dispersive spectroscopy (EDS) unit. Backscattered electron (BSE) mode and accelerating voltage of 10 and 15 kV were used for SEM observation and EDS mapping, respectively. Cross sections of all specimens (for examination with Desktop SEM) were prepared by cutting the samples in half, depositing a very thin layer of Au using a Leica EM ACE200 sputtering device, and Ni-plating using a Watts bath. Afterward, samples were mounted in epoxy resin and polished with diamond pastes. The phase composition was analyzed by the X-ray diffraction pattern by means of the X’Pert MPD diffractometer in the Bragg–Brentano configuration as well as in the grazing incidence (GID) mode. Raman imaging was performed using the WITec Alpha 300 M+ spectrometer equipped with a motorized XYZ stage, Zeiss 100× objective, 488 nm diode laser, and 600 gr/mm grating. The 3D scan was carried out on a 40 × 40 × 3 μm XYZ space. The acquisition time of each spectrum was set to 1 s. Data processing was carried out using the WITec ProjectFIVE Plus software. Spectral deconvolution was performed using Lorentz functions. X-ray photoelectron spectroscopy (XPS) was performed with a CLAM2 XPS Spectrometer (VG Microtech Ltd., London, United Kingdom). Measurements were performed in a vacuum, within the binding energy ranging from 0 to 1300 eV.

### Examination of Antiviral Activity

T4 bacteriophage (*Tevenvirinae*) and MS2 bacteriophage (*Fiersviridae*) were examined during the experiment. These phages represented the groups of viruses with different types of genome organization: double-stranded DNA (dsDNA; T4) and single-stranded RNA ((+)ssRNA; MS2). As the host for the T4 phage, the*E. coli* BL21 strain was used; for the MS2, the host was the *E. coli* C3000 strain. Each examined phage was suspended in a 1.5 mL Eppendorf tube in the TM buffer solution to reach the initial concentration of 10^6^ PFU/mL (plaque-forming units per mL, equivalent to the number of active bacteriophages in 1 mL of suspension) and the initial volume of 1 mL. Then, pieces (approximately 5 × 20 mm) of the control or coated foils were placed within the tube with bacteriophage suspension. As a control, bacteriophage suspensions without any additional material were examined. The samples were incubated for 4 h at room temperature with shaking (220 rpm). After the incubation, the titration was performed by a droplet test on double-layer LB-agar plates. The top-agar layer contained host cells of *E. coli* BL21 (T4 phage) or *E. coli* C3000 (MS2 phage). The droplet test was performed by placing at least eight droplets (5 μL each) of each phage suspension on the top-agar layer. The plates were incubated overnight at 37 °C. Then, the number of bacteriophages was calculated based on the number of the plaques according to the following equation: PFU/mL = *N* × *D* × 10 (*N:* number of plaques; *D*: dilution). The experiments were conducted in triplicate.

### Examination of Antibacterial Activity

For the evaluation of the antibacterial properties, *E. coli* BL21 strain (obtained from the collection of the Institute of Biochemistry and Biophysics PAS, Warsaw, Poland) and *S. aureus* ATCC 43300 strain (obtained from the collection of the Institute of Physical Chemistry PAS, Warsaw, Poland) were used. A colony of the required strain was picked from the stock plates and transferred to 10 mL of the LB medium to prepare the overnight bacterial cultures (37 °C, 200 rpm, using the orbital shaker–incubator ES-20). The overnight cultures were refreshed by adding fresh LB medium (1:4 v/v) and incubated at 37 °C for approximately 1 h. We aimed to reach the proper OD_600_ corresponding to the known concentration of bacteria expressed as CFU/mL (colony-forming units per mL; equivalent to the number of bacterial cells in 1 mL): for *E. coli*, OD_600_ = 1.0 => 1.0 × 10^8^, and for *S. aureus*, OD_600_ ∼1.0 => 1.5 × 10^9^ CFU/mL. Such suspensions were centrifuged at 8200 rpm for 10 min. Each bacterial culture was suspended in a 1.5 mL Eppendorf tube in 0.9% NaCl solution to reach the initial concentration of about 10^5^ cells/mL and the initial volume of 1 mL. Then, pieces (approximately 5 × 20 mm) of the control or coated foil were placed within the tube with bacterial suspension. As a control, bacterial suspensions without any additional material were used. The samples were incubated for 4 h at room temperature with shaking (220 rpm). After the incubation, 100 μL of each sample was transferred onto the fresh LB agar plates. The plates were incubated overnight at 37 °C. Then, the number of bacteria was calculated based on the colony number according to the following equation: CFU/mL = *N* × *D* × 10 (*N*: number of colonies; *D*: dilution). The experiments were conducted in triplicate.

All the experiments pertaining to antibacterial and antiphage activity were performed in biological repetitions. The number of technical repetitions for each biological replicate was at least three in the case of antibacterial verification and at least seven for antiphage experiments. The average of the obtained results was plotted on the graphs. The experiment’s standard deviations cause the error bars, as seen on the graphs.

## Supporting Information Available

SEM and EDS analysis of the cross section of the deposited (a) CuL, (b) CuL-PVP, and (c) CuL-D(+)G copper oxide layers (Figure S1); EDS chemical composition of the obtained oxide layers (Table S1); sample thickness (Figure S2); chemical composition of the layers by means of XRD-GID analysis (Table S2); Raman spectra (Figute S3); and XPS surface composition (Table S3) (PDF)
