# Supplementary material for: Copper Oxide Electrochemical Deposition to Create Antiviral and Antibacterial Nanocoatings
Source: Langmuir. 2024 Jul 9;40(29):14838–46. doi: 10.1021/acs.langmuir.4c00642 (PMC11270987; doi:10.1021/acs.langmuir.4c00642)
Supplement: Supplementary file 1 — la4c00642_si_001.pdf [file la4c00642_si_001.pdf]

Supplementary Information

Copper oxide electrochemical deposition to create antiviral and antibacterial nanocoatings

Anna Kusior[a], Julia Mazurkow[a], Piotr Jelen[a], Maciej Bik[a], Sada Raza[b], Mateusz Wdowiak[b], Kostyantyn Nikiforov[b], Jan Paczesny[b*]*

[a]AGH University of Krakow, Faculty of Material Sciences and Ceramics, Mickiewicza 30, 30-059 Kraków, Poland
[b] Institute of Physical Chemistry, Polish Academy of Sciences, Kasprzaka 44/52, 01-224 Warszawa, Poland
*akusior@agh.edu.pl, jpaczesny@ichf.edu.pl

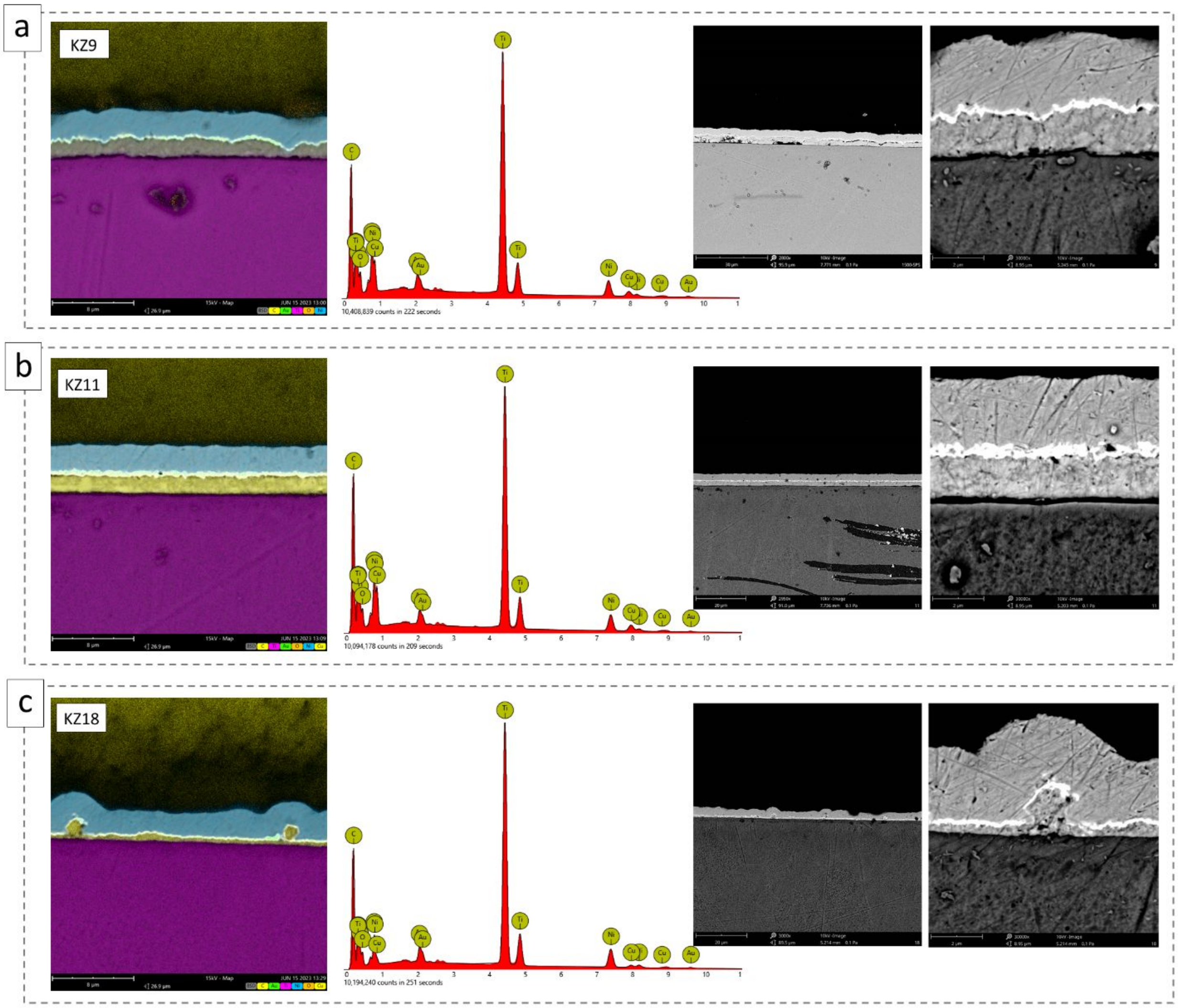

Figure S1. SEM and EDS analysis of the cross section of the deposited (a) CuL, (b) CuL-PVP, and (c) CuL-D(+)G copper oxide layers.

Table S1. EDS chemical composition of the obtained oxide layers

sample	Cu		O		Ti		Ni		Au		C	
	At%	Wt%	At%	Wt%	At%	Wt%	At%	Wt%	At%	Wt%	At%	Wt%
**CuL**	1.32	1.50	5.56	1.58	17.68	15.04	3.22	3.36	19.21	67.21	53.01	11.31
**CuL-PVP**	1.55	1.97	4.69	1.50	16.85	16.13	3.23	3.80	15.92	62.72	57.76	13.88
**CuL-D(+)G**	0.65	0.71	3.83	.105	19.05	15.62	4.07	4.09	20.08	67.76	52.31	10.76

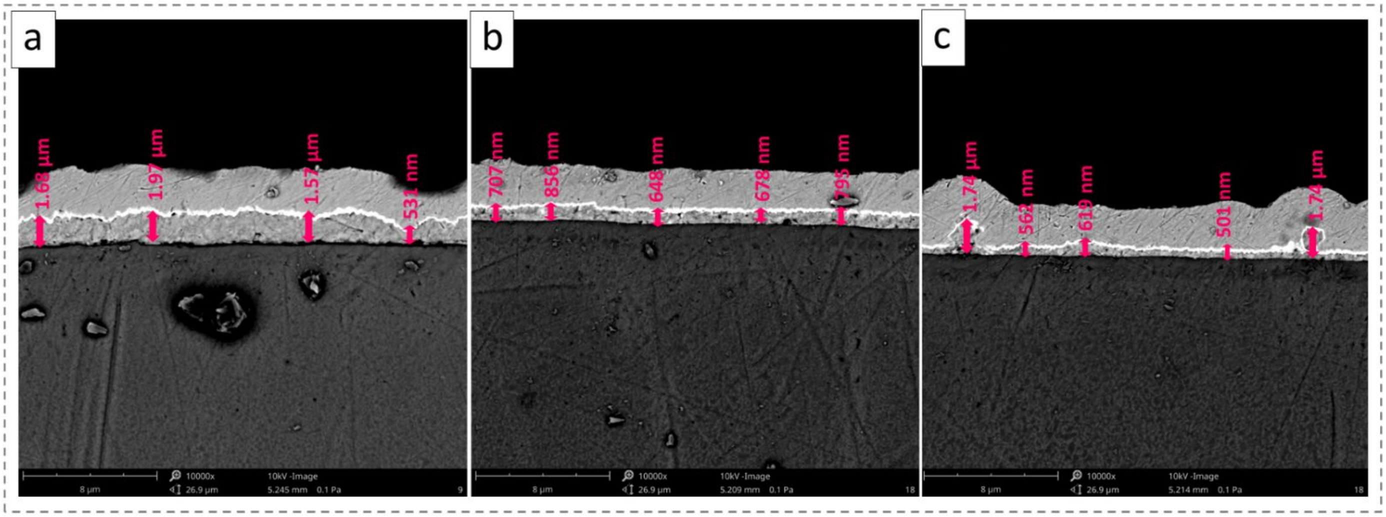

Figure S2. The sample thickness according to the SEM cross-section images of the obtained copper oxide layers.

Table S2. Chemical composition of the layers by means of the XRD-GID analysis

sample	%CuO		$\%Cu_2O$		%Ti	
	ω=0.9°	ω=2.5°	ω=0.9°	ω=2.5°	ω=0.9°	ω=2.5°
**CuL**	73.7	56.4	10.7	19.3	15.6	24.2
**CuL-PVP**	81.1	68.1	6.6	15.9	12.3	16.0
**CuL-D(+)G**	0.2	0.1	83.7	74.5	16.0	25.4

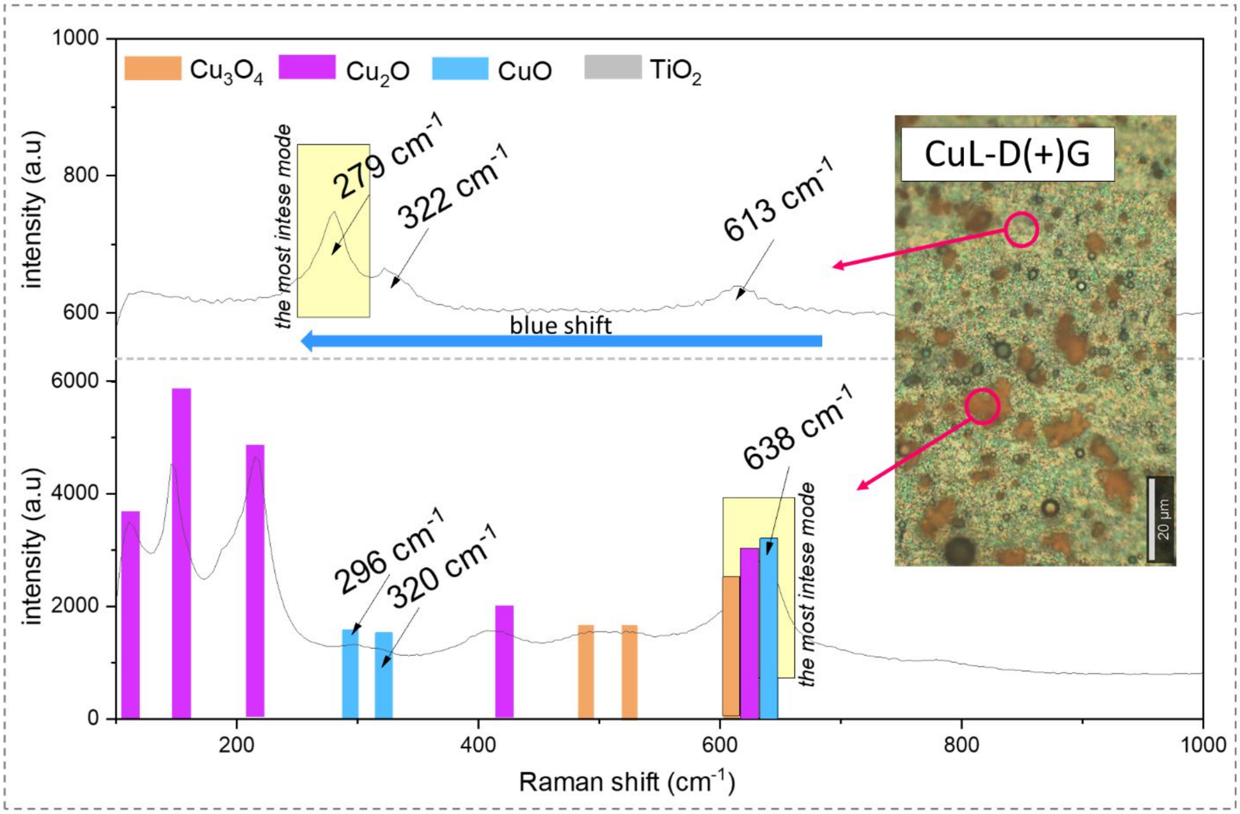

Figure S3. Raman spectra of the selected points at the CuL-D(+)G surface.

Table S3. XPS surface composition of the obtained copper oxide layers

sample		Cu 2p3/2	O 1s	S 2p3/2	S 2p1/2	Na 1s	C 1s
**CuL**	position	932.36<br>933.51<br>935.14	529.27<br>530.29<br>531.29			1071.28	284.74<br>286.13<br>288.31
	%At Conc	18.74	31.02			5.87	44.37
**CuL-PVP**	Position	932.49<br>934.66	529.86<br>531.44<br>530.48	167.46	168.65	1071.2	284.76<br>286.46<br>288.25
	%At Conc	10.8	20.51	0.52		2.71	65.46
**CuL-D(+)G**	position	932.68<br>935.16	529.60<br>530.63<br>531.70	167.75	168.95	1071.12	284.86<br>286.72<br>288.51
	%At Conc	14.75	24.87	0.77		1.29	58.33
